# The falling teacup: a curious stroke case

**DOI:** 10.1590/0004-282X-ANP-2021-0163

**Published:** 2021-12-07

**Authors:** Augusto Rachão, Tiago Geraldes, Cláudia Guarda

**Affiliations:** 1 Hospital Garcia de Orta Departamento de Neurologia Almada Portugal Hospital Garcia de Orta, Departamento de Neurologia, Almada, Portugal.

**Figure 1:**
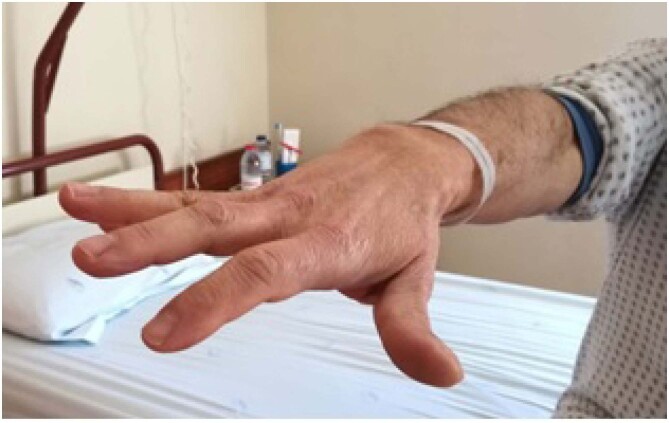
Photograph of the involved hand, evidencing a tendency toward the flexion of the right index finger, since the patient could not fully extend, adduct, and abduct it.

**Figure 2:**
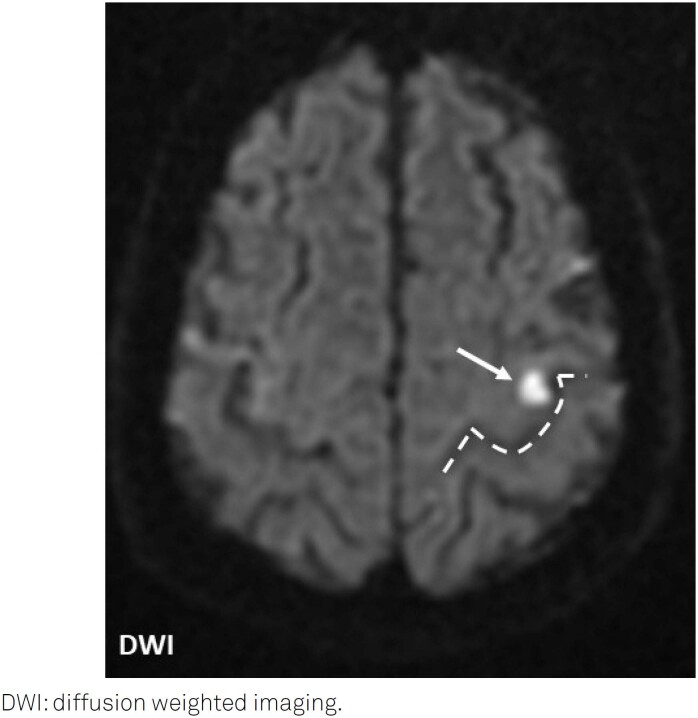
Brain magnetic resonance imaging (axial section) displaying an acute infarction (white arrow) in the hand knob territory, presenting as an inverted omega (dashed line).

A 63-year-old man presented with sudden-onset right index finger weakness that made him drop his morning teacup. He could not fully extend, adduct, and abduct his finger (Figure 1). A brain magnetic resonance imaging revealed a minor acute ischemic lesion on the left precentral cortex, in the hand knob (HK) area (Figure 2), of undetermined etiology.

HK infarctions are uncommon, often cardioembolic, and can present with different patterns of isolated hand weakness^1^. This case illustrates the rare involvement of a single finger following HK stroke and strengthens the existence of finger somatotopy in human motor cortex^1,2^. This observation helps clarifying motor cortex anatomy and clinical correlations, further detailing the Penfield’s homunculus^3^, its first proposed somatotopic organization.
